# Expression of HPV-11 L1 protein in transgenic *Arabidopsis thaliana *and *Nicotiana tabacum*

**DOI:** 10.1186/1472-6750-7-56

**Published:** 2007-09-12

**Authors:** Thomas O Kohl, Inga I Hitzeroth, Neil D Christensen, Edward P Rybicki

**Affiliations:** 1 Institute of Infectious Disease and Molecular Medicine, Faculty of Health Sciences, University of Cape Town, PO Observatory 7925, Cape Town, South Africa; 2 Department of Molecular and Cell Biology, University of Cape Town, PB Rondebosch 7701, Cape Town, South Africa; 3 Department of Pathology, The Jake Gittlen Cancer Research Institute, and Department of Microbiology and Immunology, College of Medicine, Pennsylvania State University, Hershey, Pennsylvania 17033, USA

## Abstract

**Background:**

We have investigated the possibility and feasibility of producing the HPV-11 L1 major capsid protein in transgenic *Arabidopsis thaliana *ecotype Columbia and *Nicotiana tabacum *cv. Xanthi as potential sources for an inexpensive subunit vaccine.

**Results:**

Transformation of plants was only achieved with the HPV-11 *L1 *gene with the C-terminal nuclear localization signal (NLS^-^) encoding region removed, and not with the full-length gene. The HPV-11 *L1 NLS*^- ^gene was stably integrated and inherited through several generations of transgenic plants. Plant-derived HPV-11 L1 protein was capable of assembling into virus-like particles (VLPs), although resulting particles displayed a pleomorphic phenotype. Neutralising monoclonal antibodies binding both surface-linear and conformation-specific epitopes bound the *A. thaliana*-derived particles and – to a lesser degree – the *N. tabacum*-derived particles, suggesting that plant-derived and insect cell-derived VLPs displayed similar antigenic properties. Yields of up to 12 μg/g of HPV-11 L1 NLS^- ^protein were harvested from transgenic *A. thaliana *plants, and 2 μg/g from *N. tabacum *plants – a significant increase over previous efforts. Immunization of New Zealand white rabbits with ~50 μg of plant-derived HPV-11 L1 NLS^- ^protein induced an antibody response that predominantly recognized insect cell-produced HPV-11 L1 NLS^- ^and not NLS^+ ^VLPs. Evaluation of the same sera concluded that none of them were able to neutralise pseudovirion *in vitro*.

**Conclusion:**

We expressed the wild-type HPV-11 *L1 NLS*^- ^gene in two different plant species and increased yields of HPV-11 L1 protein by between 500 and 1000-fold compared to previous reports. Inoculation of rabbits with extracts from both plant types resulted in a weak immune response, and antisera neither reacted with native HPV-11 L1 VLPs, nor did they neutralise HPV-11 pseudovirion infectivity. This has important and potentially negative implications for the production of HPV-11 vaccines in plants.

## Background

Papillomaviruses are small species- and tissue specific double-stranded DNA tumour viruses, classified in the taxonomic family *Papillomaviridae*. High-risk genital HPVs types 16, 18, 33 and 58 are the leading cause of cervical cancer [1], and low-risk genital HPVs such as the related types 6 and 11 cause benign epithelial papillomas or warts. HPV-11 is recognised as one of the most prevalent anogenital papillomaviruses and is the main causal agent of benign genital warts (condyloma acuminata) and laryngeal condylomas. Furthermore, HPV-11 DNA has also been found to be associated with various other mucosal surfaces [2-5].

Given a HPV-11 prevalence rate of 5–12% in normal women [6-8], a serious recent concern is the impact of increasing human immunodeficiency virus (HIV) infection rates, and the associated immunosuppression of HIV-positive individuals, on HPV-6 or 11 coinfections. HPV-associated disease is the most common coinfection and comorbidity in immunosuppressed individuals [9]. HPV infections are more readily detected in HIV-seropositive women, are more persistent, more severe and more difficult to treat than HPV infections in HIV-seronegative women, and recur more frequently. Silverberg *et al*. [10] have found the prevalence of HPV-6 and 11 to be up to 5.6 times higher in HIV-seropositive women, thereby increasing the prevalence of genital warts by a factor of 3.2. The associated morbidity and negative effects on quality of life are a major problem among HIV-infected women (L Denny, pers comm). Thus, although HPVs 6 and 11 are not cancer-causing, infections can be disfiguring and cause severe discomfort. The complications of HPV-11 coinfection in HIV-seropositive individuals necessitate the urgent development of a safe, efficacious and inexpensive vaccine against HPV-11.

While efforts to develop HPV vaccines have largely concentrated on the cervical cancer-causing HPV-16, HPV-11 has also received widespread attention. Extremely high healthcare costs are associated with management of non-cancerous HPV-6/11 disease, and inclusion of HPV-6 and 11 in a vaccine might be advantageous [11]. Phase I clinical trials have proven that HPV-11 L1 virus-like particle (VLP) vaccines are safe [12]. Merck has developed a quadrivalent HPV vaccine, Gardasil™, which includes HPV-6, 11, 16 and 18 and is produced in yeast: two large Phase III trials have been completed and it was licensed for use in the United States in June 2006 [13]. A modelling study [14] predicted significant improvements in the quality of life and prevention of cancer upon vaccination of young girls with an HPV vaccine that was only 75% efficacious. However, the Merck HPV vaccine will cost US$360 for 3 doses/person – an amount that is higher than the annual per capita health expenditure of many third-world nations. Therefore, other strategies for the production of stable, cheaper HPV vaccines are more immediately appropriate for these constituencies.

A strategy for the large-scale production of inexpensive HPV vaccines is production in plants: this could be between 10 and 50 times cheaper than its production in fermentation systems. Delivery of these by the oral or "needle-free" route is ideally suited to the background setting of vaccination campaigns in many developing countries [15]. We and others have shown that plant-expressed HPV-11 L1 and HPV-16 L1 proteins assemble into antigenically-appropriate capsomers and VLPs that are highly immunogenic upon parenteral and/or oral delivery to animals [16-20]. HPV-11 VLPs were expressed in potato; however, yields were very low and oral administration of tuber material was only weakly immunogenic [19]. We have also shown that a plant-produced cottontail rabbit papillomavirus (CRPV) capsomer vaccine protected rabbits against the formation of CRPV-induced papillomas [21], which so far is the only proof of concept for a plant-produced PV L1 protein vaccine.

This study aimed to develop a plant-derived L1 protein subunit vaccine against HPV-11 in a plant host from which it could be easily purified at reasonable yield, in order to formulate a potentially cheap vaccine that could help prevent HPV-11 infection in developing countries. Through *Agrobacterium tumefaciens*-mediated transformation, we genetically manipulated *Arabidopsis thaliana *and *Nicotiana tabacum*, to express the HPV-11 *L1 *gene. Comparative studies on the expression of the gene were performed on both expression systems. Products were characterised antigenically using monoclonal antibodies and used to immunise rabbits, in parallel with insect cell-produced antigens.

## Results

### Genetic analysis of transgenic plants

Despite several attempts to transform *A. thaliana *and *N. tabacum *plants with the HPV-11 *L1 *and HPV-11 *L1 NLS*^- ^genes, only plants containing the HPV-11 *L1 NLS*^- ^gene could be regenerated. Attempts to introduce the full-length HPV-11 *L1 *gene failed in both plant systems. PCR screening of putative HPV-11 *L1 NLS*^- ^transgenic *A. thaliana *first generation (T_1_) and *N. tabacum *regenerated generation (R_0_) plants, using primer pair 4 as listed in Table 1, showed that 8/20 *A. thaliana *T_1 _generation and 4/15 *N. tabacum *R_0 _lines were positive for the gene.

**Table 1:** Primers used for the amplification of the HPV-11 *L1 *and *L1 NLS*^- ^ORFs

**Primer Pair**	**Construct**	**Primers**
1	pHPL51 (502)	Forward 5'- GGAATTCCGGAGGAATTTTTTACAG**ATG**TGGCGGC -3'
		Reverse 5'- GACGCCGTCGACTCCGGAACTGACACACATATA**TTA**G -3'
2	pHPS51 (ΔC481)	Forward 5'- GGAATTCCGGAGGAATTTTTTACAG**ATG**TGGCGGC -3'
		Reverse 5'- ACGCCGTCGACTCCGGA**TTA**TATACCTGTTACGAGCAGC -3'
3	pART27 *L1*	Forward 5'- **ATG**TGGCGGCCTAGCGAC -3'
		Reverse 5'- **TTA**CTTTTTGGTTTTGGTACGTTTTCG-3'
4	pART27 *L1 NLS*^-^	Forward 5'- **ATG**TGGCGGCCTAGCGAC -3'
		Reverse 5'- **TTA**TATACCTGTACGAGCAGACG -3'
5	HPV-11 *L1 NLS*^- ^internal	Forward 5'- TGATACAGGCTTTGGTGC -3'
		Reverse 5'- TGGCTCATTGGTGTCTTC -3'

GAATTC:*Eco*RI; TCCGGA:*Mro*I, GTCGAC:*Sal*I, **ATG** = start codon, **TAA** = stop codon

**Figure 1:**
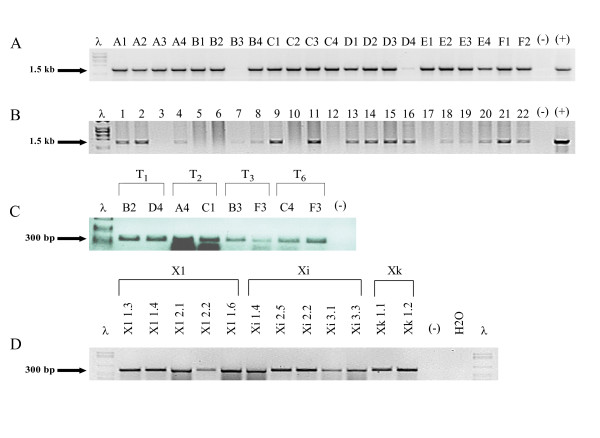
**Genomic DNA analysis of transgenic plants**. (**A**) Analysis of individual transgenic *A. thaliana *lines of the T_6 _generation (A1-4 represent 4 plants of line A; B1-4 plants from line B etc.), indicating the presence of the HPV-11 *L1 NLS*^- ^gene. (**B**) Screening of genomic DNA extracted from transgenic *N. tabacum *line X1 (T_1 _generation) plants for the presence of the HPV-11 *L1 NLS*^- ^gene. The majority of the individual plants are positive for the HPV-11 *L1 NLS*^- ^gene, as is shown by a 1.5 kb PCR amplification product. Genomic DNA extracted from respective non-transgenic plants and HPV-11 *L1 NLS*^- ^plasmid DNA represent the negative and positive controls respectively. (**C**) RT-PCR analysis of PCR positive transgenic *A. thaliana *plants from the T_1_, T_2_, T_3 _and T_6 _generations and (**D**) of individual T_1_, T_2 _and T_3 _generation *N. tabacum *plants from lines **X1**, **Xi**, and **Xk**. (for example: **Xi 2.5 **represents line **Xi**, T_2 _generation, plant number 5). Individual plants from the respective lines express the *L1 NLS*^- ^gene as shown by the 303 bp amplification product. Total RNA extracted from respective non-transgenic plants represent the negative control.

Inheritance of the transgene was established by self-pollinating T_1 _plants and screening seeds on selective media. Random PCR screening of plant lines up to the T_6 _generation for *A. thaliana *and T_3 _generation for *N. tabacum *showed that the gene was stably integrated and successfully inherited (Figure 1A and 1B).

To determine transcription of the integrated HPV-11 *L1 NLS*^- ^gene, total RNA of successive generations of *A. thaliana *and *N. tabacum *plants was analysed by RT-PCR. Amplification of a 303 bp product indicated the presence of the HPV-11 *L1 NLS*^-^transcript and we determined that the HPV-11 *L1 NLS*^- ^mRNA was present in PCR-positive transgenic plants in all generations (Figure 1C and 1D).

### Analysis of plant-derived HPV-11 L1 NLS^- ^protein

**Figure 2:**
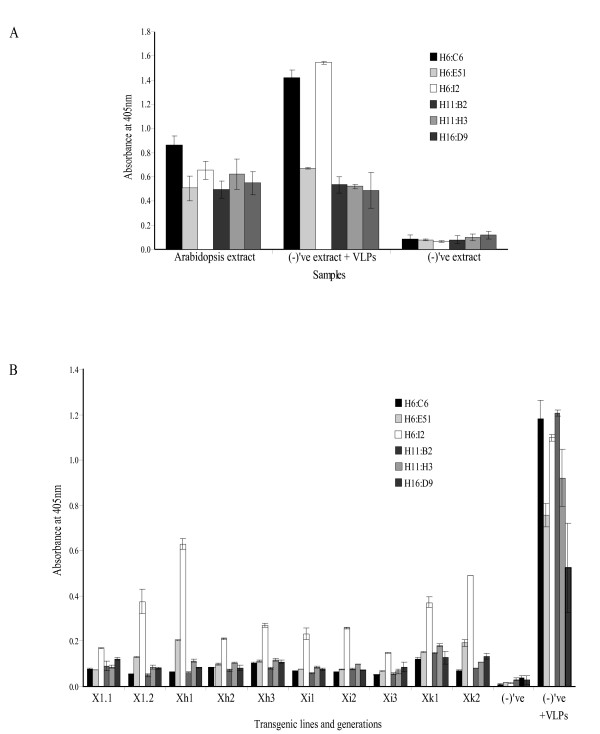
**Detection of HPV-11 L1 protein in plant extracts using ELISA**. (**A**) VLP characterisation by ELISA, utilising conformation-specific and linear MAbs, of HPV-11 L1 NLS^- ^transgenic T_3 _generation *A. thaliana *protein extract from pooled lines and (**B**) *N. tabacum *protein extract from individual lines and generations. (for example: X1.2 represents line **X1**, T_2 _generation). Non-transgenic *A. thaliana *or *N. tabacum *protein extract was used as negative control; the positive control consisted of non-transgenic protein extract spiked with 0.4 μg/well insect cell-derived HPV-11 L1 NLS^- ^VLPs. Error bars represent the standard deviation calculated from triplicate analysis of samples.

**Figure 3:**
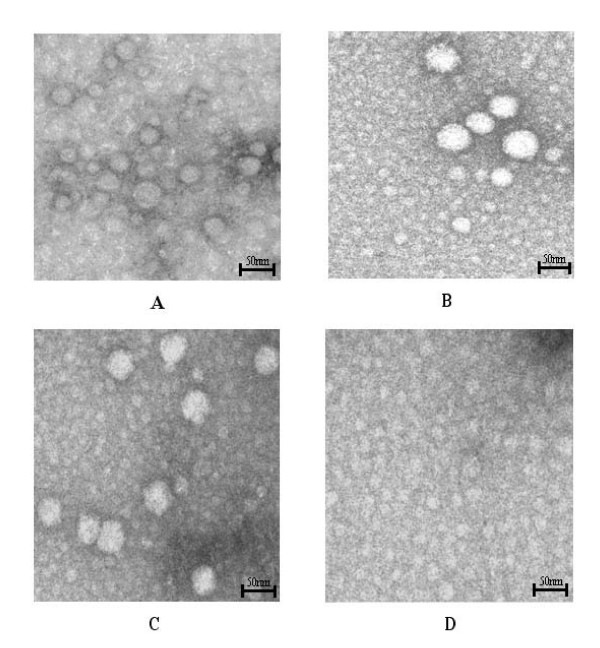
**Electron microscopy of plant extracts**. (**A**) Electron micrograph of T_3 _generation *A. thaliana*-derived HPV-11 L1 NLS^- ^VLPs. (**B**) Electron micrograph of *N. tabacum*-derived HPV-11 L1 NLS^- ^VLPs. Particles were immunotrapped using polyclonal antiserum at a dilution of 1:50 and on average range from 30 to 60 nm in diameter. (**C**) The positive control shows H11;H3 immunotrapped insect cell-derived HPV-11 L1 NLS^- ^VLPs, whereas immunotrapped non-transgenic plant protein extract is represented in **D**.

The final product resulting from homogenization, filtration and centrifugation of transgenic leaf material was analysed by standard techniques and showed that HPV-11 L1 NLS^- ^protein was indeed present in the concentrated plant protein extracts (Figures 2 and 3).

Monoclonal antibodies (Mabs) to HPV-6 (H6:C6, H6:E51 and H6:I2); HPV-11 (H11:B2 and H11:H3) and to HPV-16 (H16:D9) displaying specific and cross-reactive properties (Table 2) were used in the ELISA to detect and characterise the antigenicity of HPV-11 L1 NLS^- ^protein derived from both plant hosts (Figure 2A and 2B). For *A. thaliana*-derived HPV-11 L1 NLS^- ^protein, T_3 _generation plants from all 8 lines were harvested and pooled. Our initial interest was in determining whether or not the protein was expressed at all in *A. thaliana*, rather than in selecting high or low expressers. The limited biomass obtainable from these plants necessitated the pooling of leaf material from all transgenic lines. The resulting protein extract was characterised with all the above listed Mabs. The results in Figure 2A show that all Mabs bound to the *A. thaliana*-derived L1-containing protein extract. Binding of HPV-11 neutralising conformation-specific Mabs H11:B2 and H11:H3 to antigen in the plant protein extract was essentially equivalent to the binding to the positive control, suggesting that *A. thaliana*-derived HPV-11 L1 NLS^- ^protein could potentially elicit a neutralising antibody response once administered.

**Table 2:** MAbs used for the detection and characterisation of the HPV-11 L1 NLS^- ^protein

**Monoclonal antibody**	**Isotype**	**Neutralisation**	**Nature of Epitope**	**Cross-reactivity**	**References**
H11:B2	IgG2b	Yes (HPV-11)	conformation-specific	Yes (HPV-6 & 11)	[13] [34] [35] [36]
H11:H3	IgG2b	Yes (HPV-11)	conformation-specific	No	
H6:C6	IgG2a	No (HPV-11)	linear (surface)	Yes (HPV-6 & 11)	
H6:E51	IgG1	No (HPV-11)	linear (surface)	Yes (HPV-6 & 11 & 18)	
H6:I2	IgM	Yes (HPV-11)	linear (surface)	Yes (HPV-6 & 11)	
H16:D9	IgG1	No (HPV-11)	linear (surface)	Yes (HPV-11 & 16)	

Further, Figure 2A shows evidence of binding of the HPV-6/11 cross-reactive surface linear Mabs H6:C6, H6:E51 and H6 I2 to the protein extract: these Mabs bind intact VLPs as well as denatured L1 protein. Although binding of these Mabs was not of the same magnitude as to the positive control (Figure 2A), together these results suggest that most of the *A. thaliana*-derived HPV-11 L1 NLS^- ^protein is assembled similarly to insect cell-produced protein.

Analysis of the antigenic properties, of the *N. tabacum*-derived HPV-11 L1 NLS^- ^protein from all generations from 4 lines showed that the surface linear and HPV-11 neutralising antibody H6:I2 bound best. Two other cross-reactive Mabs (H6:C6 and H6:E51) also bound; however, binding efficiencies of these surface exposed linear epitope-recognizing and non-HPV-11-neutralising Mabs were significantly lower (Figure 2B).

Conformation-specific and HPV-11 neutralising Mabs H11:B2 and H11:H3, which only bind capsomers and intact VLPs, did not react significantly with plant extracts, suggesting that most of the *N. tabacum*-derived HPV-11 L1 NLS^- ^protein exists in an unassembled state. The rather weak detection of the HPV-11 L1 NLS^- ^VLP spiked positive control by MAb H16:D9 was anticipated, as it is not suitable for detecting intact VLPs (Figure 2B).

A similar assay on insect cell-derived HPV-11 VLPs diluted in PBS instead of non-transgenic plant extract gave qualitatively identical results to those in Figures 2A and 2B (result not shown), indicating that addition of plant sap does not change the VLP antigenic properties.

### Quantitation of the L1 protein

The amount of L1 protein in transgenic plant extracts was measured by comparison to an ELISA-derived standard curve for known concentrations of insect cell-derived HPV-11 L1 NLS^- ^protein. Non-transgenic plant protein extract was initially spiked with insect cell-derived HPV-11 L1 NLS^- ^VLPs resulting in a known concentration of 0.4 μg per well (100 μl); O.D._405nm _comparisons allowed calculation of the total amount of L1 protein from transgenic *A. thaliana *and *N. tabacum *extracts for which the total plant weight and homogenisation buffer volume was known. Overall yields of L1 protein harvested from *A. thaliana *were calculated to range from between 3 and 12 μg/g, whereas *N. tabacum *plants yielded between 0.2 and 2.2 μg/g of fresh leaf material.

### Electron microscopy

*A. thaliana *and *N. tabacum *protein extracts were immunotrapped onto grids using anti-HPV-11 L1 polyclonal antiserum and stained with 2% uranyl acetate (Figures 3A and 3B). A range of different particle sizes varying from 20 to 60 nm in diameter were observed in both protein extracts. Furthermore, the presence of L1 protein was detected by immunogold-labeling of the plant-derived extracts, thus reconfirming these observations (results not shown).

Insect cell-produced VLPs in non-transgenic plant extract immunotrapped using Mab H11:H3 represent the positive control (Figure 3C); negative controls were protein extracts derived from non-transgenic *A. thaliana *(not shown) and *N. tabacum *(Figure 3D): these showed no HPV-like or any other particles upon examination.

### Western blotting

**Figure 4:**
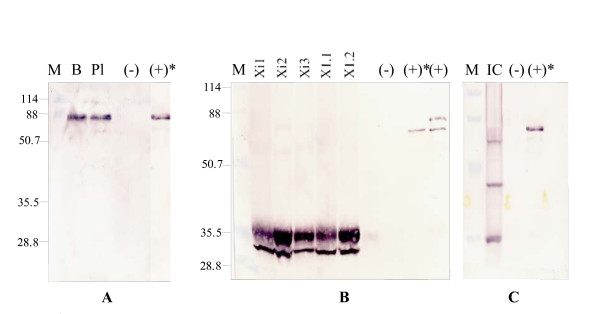
**Western blot analysis of plant extracts**. (**A**) Western blot analysis of TCA-precipitated *A. thaliana *protein extract detected with H6:E51. HPV-11 L1 NLS^- ^protein is present in both protein extracts from primary bolts and complete plants. M = MW marker; B = bolts; Pl = plants. (**B**) Western blot analysis of TCA precipitated *N. tabacum *protein extract detected with H6:I2. The HPV-11 L1 NLS^- ^protein is present in all generations of the *N. tabacum *transgenic lines and is ~36 kDa in size. (-) represents the TCA precipitated non-transgenic control. The positive controls (+) and (+)* are insect cell-derived CsCl-purified HPV-11 L1 (NLS^+^) and HPV-11 L1 NLS^- ^VLPs, respectively. (**C**) Western blot analysis of non-concentrated, non-purified HPV-11 L1 NLS^- ^VLPs in insect cell lysate detected with H6:C6 (IC). Non-infected insect cell lysate was used as negative control (-) in **C**.

Detection of HPV-11 L1 NLS^- ^protein by western blot is shown in Figure 4. While characteristic polypeptide bands were detected in both plant extracts, the L1 protein extracted from *A. thaliana*-derived L1 protein showed no proteolysis: a ~55 kDa band in (A) matched that of the positive control. In contrast, however protein from *N. tabacum *(B) was extensively proteolysed (~36 kDa) indicating that protease activities differ markedly between the 2 plant systems. HPV-11 L1 NLS^- ^in insect cell lysate detected with H6:C6 (shown in C as Insect cell (IC)) was cleaved twice, resulting in 3 products: one of these is ~36 kDa in size. Non-infected insect cell lysate was used as negative control in C.

### Animal immune response to plant-derived HPV-11 L1 protein

**Figure 5:**
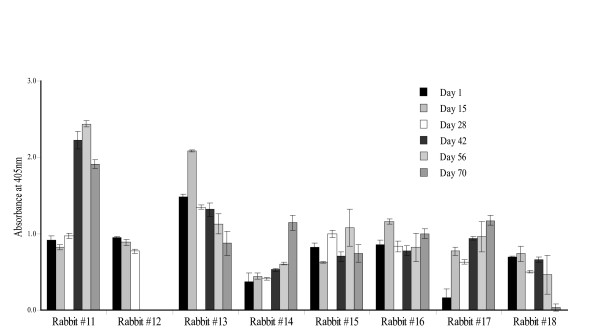
**Immunogenicity of plant extracts**. Analysis of serum from New Zealand white rabbits #11 and #12 immunized with *A. thaliana*-derived HPV-11 L1 NLS^- ^VLPs and rabbits #13 to #18 immunized with *N. tabacum*-derived HPV-11 L1 NLS^- ^VLPs. Rabbits were immunized on days 1, 28 and 56 and serum from days 1, 15, 28, 42, 56 and 70 post injection was tested by ELISA in wells coated with 0.4 μg insect cell-derived HPV-11 L1 NLS^- ^VLPs. Data from rabbit #12 is incomplete as it had to be euthanized due to growth of an abscess on the neck. Error bars represent the standard deviation calculated from triplicate analysis of samples.

Eight New Zealand white rabbits were immunized with concentrated transgenic *A. thaliana *and *N. tabacum *protein extracts. Serum collected on days 1, 15, 28, 42, 56 and 70 was analysed for anti-HPV-11 L1 reactive antibodies by ELISA and results are shown in Figure 5. Rabbits #11 and #12 were immunised with T_3 _generation transgenic *A. thaliana*-derived L1 protein extract calculated to contain between 21 μg and 43 μg.

A distinct difference was noted in the immune responses of these 2 rabbits: serum from rabbit #11 reacted well with insect cell-produced NLS^- ^VLPs (Figure 5), while rabbit #12 showed no significant antibody response and can hence be classified as a non-responder.

Rabbits #13 – #18 were immunised with *N. tabacum*-derived protein extract: two rabbits per group were immunised with *N. tabacum *protein extract from T_1 _generation transgenic lines X1, Xh and Xi respectively. According to the range of protein yield calculated per kg of fresh leaf material, inoculum from line X1 contained between 13–47 μg of L1 protein, whereas inocula from lines Xh and Xi contained between 10–114 μg and 9.5–42 μg of L1 protein respectively.

Only sera from rabbits #14 and #17 reacted with HPV-11 L1 NLS^- ^VLPs (Figure 5). This did not correlate with inoculum dose, as responder #14 received 13–47 μg, non-responders #15 and #16 received 10–114 μg, and responder #17 received 9.5–42 μg. Antisera from #14 reacted 3-fold stronger in comparison to the prebleed and sera from #17 showed the highest response with antiserum reacting 7-fold stronger in comparison to prebleed antiserum.

The most interesting result was obtained by evaluating serum reactivities in ELISA against insect cell-produced HPV-11 L1 NLS^+ ^VLPs. Serum from rabbits inoculated with *A. thaliana*-derived L1 protein extract failed to react, whereas sera from rabbits#16 and #17 immunised with *N. tabacum*-derived L1 protein appeared to weakly bind this antigen, as indicated by rising absorbances of successive bleeds about two fold higher than the prebleed level (results not shown). This lack of recognition of the HPV-11 L1 NLS^+ ^VLPs is of great concern because these VLPs most closely resemble the actual virus: the implications of this will be discussed later.

**Figure 6:**
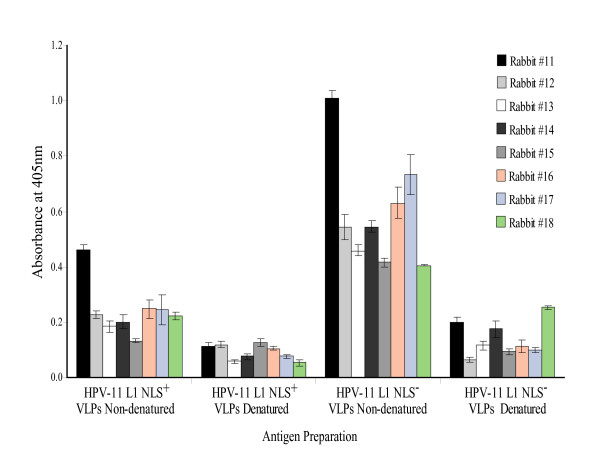
**Antigenic specificity of immune sera**. Analysis of **Day 56 **antiserum from individual rabbits immunized with HPV-11 L1 NLS^- ^plant protein extract. Sera were tested against equal concentrations of denatured and non-denatured insect cell-derived HPV-11 L1 NLS^+ ^and NLS^- ^VLPs at a concentration of 0.4 μg/well. Error bars represent the standard deviation calculated from triplicate analysis of samples. Insect cell-derived VLPs were denatured at 100°C for 10 min. Rabbits #11 and #12 were immunized with *A. thaliana*-derived HPV-11 L1 NLS^- ^antigen. Rabbits #13 and #14, #15 and #16, and #17 and #18 were inoculated with *N. tabacum*-derived protein extract from T_1 _generation transgenic plant lines X1, Xh and Xi respectively.

To further investigate this, Day 56 antisera were tested against denatured and non-denatured insect cell-derived HPV-11 L1 NLS^+ ^and NLS^- ^VLPs by ELISA. The results shown in Figure 6 essentially reconfirm those presented in Figure 5. With the exception of rabbit #11, the antisera from all remaining rabbits immunized with either plant-derived protein extract reacted predominantly with non-denatured insect cell-derived HPV-11 L1 NLS^- ^VLPs, and not with denatured protein from either type of VLPs. This was confirmed by western blots of the insect cell-derived NLS^+ ^and NLS^- ^protein. While very strong reactions were seen with baculovirus and *Sf*21 cell proteins in both prebleed and last bleed sera, neither L1 protein was detected (data not shown). This suggested that the predominant antibody response was most probably not targeted against any linear epitopes.

### Pseudovirion neutralisation assays

All rabbit sera taken on days 1 and 56 were tested for their capacity to neutralise HPV-11 pseudovirions. Serum sample dilutions ranging from 1:50 to 1:12150 were incubated together with HPV-11 L1 pseudovirions before being assayed. One of two internal controls was serum from rabbit #19 (not previously shown), which was inoculated with insect cell-derived HPV-11 L1 NLS^+ ^VLPs. Dilutions of the conformation-specific and neutralising MAb H11:H3 were the second positive control.

No specific neutralising capacity was observed for any prebleed serum. However, while rabbit #19 serum (internal positive control; anti-insect cell-produced L1) neutralised the pseudovirion at the highest dilution tested (1:12150), and the known conformation-specific and neutralising MAb H11:H3 worked to a dilution of 1:10^6^, none of the post-inoculation day 56 sera were neutralising, even at the lowest dilution of 1:50.

Taken together, these serological results indicate that plant-derived HPV-11 L1 NLS^- ^VLPs are modified to some extent, yet still retain certain overall virion morphological features as seen by electron microscopy. Immunisation of animals, however, results in an antibody response that is not capable of recognizing or neutralising HPV-11 L1 NLS^+ ^VLPs, the closest counterpart to the actual virus.

## Discussion

The focus of this study was on the expression of a native HPV-11 *L1 *gene, as full-length and *NLS*^- ^constructs, in transgenic *A. thaliana *and *N. tabacum*, with the overall aim of gaining some insight as to the feasibility of developing a plant-derived vaccine against HPV type 11. This is against a background of the release in 2006 of the GARDASIL™ yeast-derived quadrivalent vaccine against HPV types 6, 11, 16 and 18 by Merck: this vaccine has already shown 100% effectiveness in protecting from infection. However, the high cost of one course of this vaccine (3 doses; US$360) means that it will not prevent the continuous spread of the virus-associated disease in the foreseeable future, because it will remain largely non-affordable to people in disadvantaged regions of the globe.

Here we report on the successful transformation of both *A. thaliana *and *N. tabacum *with the HPV-11 *L1 *gene missing the C-terminal nuclear localisation signal (NLS). This increases the number of plant systems used for the successful expression of HPV L1 proteins to include not only tobacco and potato, but now also *Arabidopsis*. Overall, our tobacco lines yielded ~2 μg of L1 protein per g of fresh leaf material, whereas up to 12 μg/g of L1 protein could be extracted from whole *Arabidopsis *plants. Our group has previously expressed unmodified CRPV *L1 *and HPV-16 *L1 *genes in transgenic tobacco to levels of 1 μg/g and 4 ng/g of plant tissue respectively [21,17], and HPV-16 L1 via a tobamovirus vector in *N. benthamiana *to ~40 ng/g [18]. Biemelt *et al*. [16] expressed a human codon-optimised HPV-16 *L1 *gene in tobacco at a level of ~0.5% of the total soluble protein content (TSP), and in potato to about 0.2% TSP or ~12 μg/g of tuber. Warzecha *et al*. [19] expressed a plant codon-optimised HPV-11 *L1 NLS*^- ^gene in potato and reported extracting up to ~23 ng/g of fresh tuber. The HPV-16 L1 levels of expression have since been far surpassed by Maclean *et al*. [20]; this work reports a 500 – 1000 fold increase in yield compared to previous reported expression of HPV-11 L1 [19].

We found that *in planta *expression of the wild-type HPV-11 *L1 *gene presented great difficulties and that it was effectively impossible to transform either one of our host plants. Similar observations were made by Warzecha *et al*. in 2003 [19] and despite their use of a plant codon-optimised HPV-11 *L1 NLS*^+ ^gene, the number of putative transgenic plant lines remained low. The low number of confirmed HPV-11 L1 NLS^- ^transgenic plant lines in this study reiterates the observation that integration and/or expression of the HPV-11 *L1 *gene (full-length vs truncated/wild-type vs codon-optimised) is generally not well tolerated in plants.

This stands in direct contrast to results obtained with the HPV-16 *L1 *gene, where expression of full-length *L1 *in either native (Varsani et al., 2003d) or human codon-optimised forms [16,20] was readily achieved. The latter studies concluded that the human codon-optimised HPV-16 *L1 *gene expressed better in plants than the plant codon-optimised or wild-type genes. Although HPV-11 and 16 *L1 *genes differ significantly, their L1 proteins are functionally very similar. Thus, the reasons for the difference in transformability remain obscure. It is also interesting in light of the HPV-16 results that our native HPV-11 *L1 *gene expressed so much better than the plant codon-optimised candidate of Warzecha et al. [19]: this indicates that expression of HPV *L1 *genes is best assessed empirically, rather than by assuming that gene optimisation according to plant codon usage will automatically improve expression.

The NLS of the HPV-11 *L1 *gene is not required for correct VLP assembly [22]. Resulting particles remain morphologically and antigenically identical to native virions despite removal of 21 aa from the C-terminus [19]. Therefore the truncated HPV-11 L1 protein should still be usable as a vaccine and given the difficult and unpredictable *in planta *expression levels achieved with a spectrum of HPV *L1 *genes, we had decided to focus on maximizing the expression of the wild-type HPV-11 *L1 NLS*^- ^gene in our two expression hosts.

We were able to show successful transgene integration as well as stable inheritance in consecutive generations (Figure 1A and 1B). Transcription of the gene was investigated using RT-PCR: active transcription was shown throughout successive generations of both plant systems (Figure 1C and 1D). One possible shortcoming of this approach however, given that Biemelt *et al*. [16] and Warzecha *et al*. [19] found by northern blot that their respective *L1 *mRNAs were somewhat degraded, was that such degradation was not detectable in our system.

We further investigated if the L1 monomers formed VLPs. We found that expression of the HPV-11 *L1 NLS*^- ^gene via recombinant baculovirus in *Sf*21 cells resulted in a range of different sized particles that did not resemble the classical shape (Figure 3C). Furthermore, particles seen in both *A. thaliana *and *N. tabacum *protein extracts had shown the same pleomorphy, ranging from 20 to 60 nm in diameter (Figure 3A and 3B). Li *et al*. [23], previously found that removal of 11 carboxyl-terminal amino acids still allowed for VLP assembly, but that particles were less uniform than those resulting from expression of the full-length *L1 *gene. Warzecha et al. [19] found particles in HPV-11 *L1*-transformed potato but did not report pleomorphy as seen here.

Given several observations of differences between plant- and insect cell-produced HPV-11 L1 proteins, we determined whether proteolytic degradation occurred for insect cell-derived L1 protein. Crude insect cell lysate containing HPV-11 L1 NLS^- ^protein was left standing at 4°C for several hours before being examined by western blot: 3 distinct L1 proteolytic products were seen (Figure 4C), of ~53, ~45 and ~36 kDa relative to the purified 55 kDa positive control. Li *et al*. [23] had reported that the HPV-11 L1 protein is susceptible to trypsin digestion, resulting in two distinctive products of ~42 and ~48 kDa in size. Our western blot analyses of insect cell and plant-derived HPV-11 L1 NLS^- ^proteins showed that the *N. tabacum*-expressed HPV-11 L1 NLS^- ^protein is subject to severe degradation: digestion by a presumably trypsin-like protease resulted in a product of ~36 kDa (Figure 4B). Unlike proteolysis in the crude insect cell lysate (Figure 4C), no intermediate species were observed in the *N. tabacum *protein extract, suggesting that despite the addition of complete protease inhibitor throughout the extraction process, complete proteolysis of the L1 protein occurred over time.

Little is known about the properties of L1 proteins that have undergone this degree of proteolysis. Assuming they are incapable of assembly into capsids or of exhibiting virus-specific conformational epitopes could partially explain the ELISA results for tobacco-derived proteins shown in Figure 2B. However, particles were observed by electron microscopy (Figure 3B). It could be that VLP assembly occurred during the concentration process, rather than *in planta*; and then only of a subset of L1 monomers that had not yet been completely proteolysed. Although analysis of the *A. thaliana *protein extract was anticipated to yield similar results, surprisingly no degradation was observed in the extracted HPV-11 L1NLS^- ^protein (Figure 4A). Furthermore, no significant alterations to the *N. tabacum*-derived HPV-16, the potato-derived HPV-11 L1 protein or of plant-derived CRPV L1 had been reported in previous studies [16-19][21]. However, ours was the only study with HPV-11 L1 expressed in tobacco – and given the already proven unpredictability of L1 expression in plants, perhaps this is just more proof that empiricism is the only way to approach this subject.

The phenomenon of the HPV-11 L1 NLS^- ^proteins being severely degraded in *N. tabacum *plants but not in *A. thaliana *is interesting, and would undoubtedly impact on their vaccine potential. Given the evidence of proteolysis and particle pleomorphism, we investigated whether the antigenic properties of the plant-derived VLPs had been retained. It has been reported that removal of 86 aa from the HPV-11 L1 C-terminal end resulted in the formation of pentameric capsomers that were no longer able to establish inter-capsomeric contacts: however, the MAb H11:H3 capsid-neutralising domain was entirely contained within pentameric L1 capsomers and interpentamer associations were not required for the induction of virus-neutralising antibodies [24]. However, tobacco-produced VLPs did not bind the conformation-specific MAb H11:H3 (Figure 2B). Another conformation-specific and neutralising MAb (H11:B2) also failed to bind the tobacco plant-derived protein. This is similar to the case of Chen *et al*. [25], where the formation of VLPs from severely truncated COPV L1 protein did not necessarily preserve conformation-specific epitopes on the surface of the capsid. In contrast, all Mabs, including the conformation-specific and neutralising Mabs, were capable of binding to the non-proteolysed *A. thaliana*-produced HPV-11 L1 NLS^- ^(Figure 2A).

It is of utmost importance to establish whether any potential viral vaccine is capable of eliciting an appropriate antibody response. Accordingly, we evaluated the ability of the plant extracts to induce an antibody response in rabbits. With perhaps the exception of serum collected from rabbit #17 all immune sera reacted with insect cell-derived NLS^- ^VLPs (Figures 5 and 6), and not with NLS^+ ^VLPs. This was unexpected, given that especially the *A. thaliana*-derived L1 protein reacted with Mabs specific for conformation-dependent epitopes. Additionally, serum collected on day 56 reacted exclusively with non-denatured insect cell-derived NLS^- ^VLPs (Figure 6). Taken together, these results present a major problem – both for interpretation and potentially for the prospect of HPV-11 plant-produced vaccines. We note that Warzecha *et al*. [19] found that their plant-derived material reacted with appropriate antibodies, but that no reaction could be shown by sera from orally immunized mice with insect cell-derived VLPs – presumably NLS^+ ^– unless the mice had been orally boosted with those VLPs. In contrast, it has been shown that injection of plant-produced HPV-16 and CRPV L1 elicited antibodies reactive with native VLPs produced in insect cells [16,17,20,21]. Thus, this phenomenon is apparently limited to the HPV-11 L1 NLS^- ^protein.

Of more concern to the prospects of a plant-produced HPV-11 vaccine was the fact that none of the sera collected on day 56 could neutralise HPV-11 pseudovirions. While we have previously shown that rabbits immunized with plant-derived CRPV L1 protein were protected against live CRPV challenge in the absence of detectable neutralising antibodies [21], this is the only such result to our knowledge, and it is generally taken as given that the primary correlate of protection against papillomavirus infection is the presence of neutralising antibodies. We note too that Maclean *et al*. [20] were able to show pseudovirion neutralisation after immunisation of mice with plant-derived HPV-16 L1 VLPs.

We suggest that an immune response to plant-derived HPV-11 L1 NLS- is elicited mainly against an immunodominant epitope that appears to be surface exposed in VLPs. The nature of the structural changes that occur within the capsid that allow for recognition of this epitope, which is otherwise not surface exposed on the actual HPV-11 virion, need further investigation. Nevertheless, the lack of recognition by immune sera of the HPV-11 L1 NLS^+ ^VLPs, the closest counterpart to the actual virus, implies that antibodies generated from injection of this particular plant-derived HPV-11 L1 NLS^- ^protein will not be able to prevent infection and disease, which completely negates their potential use for prophylactic vaccination.

However, we feel it would be a mistake to neglect further work on the development of an HPV-11 vaccine in plants. With the possibility of using a plant-derived HPV-11 vaccine as cost-effective vaccine boosters, the goal would be to make it work effectively. It might be possible to do this by engineering the protein in a different way, or making HPV-11:HPV-16 hybrid L1s, for example. It will also be interesting to investigate if with new second and third generation plant expression vectors, expression of full length native HPV-11 L1 protein can be achieved, and if alternative codon optimisation or expression strategies will have an impact on expression levels, conformation and immunogenicity of this product.

## Conclusion

In summary, we expressed the HPV 11 *L1 NLS*^- ^gene in two different plants, upon which we found that in *A. thaliana *VLPs were formed. These particles were recognised by conformation-specific antibodies and when analysed by western blot, a 55 kDa protein was observed. In contrast, however, HPV 11 L1 NLS^- ^VLPs from *N. tabacum *were not recognized by the same conformation-specific antibodies even though VLPs were observed by EM. The protein seemed to be partially degraded as shown by the presence of a 36 kDa band in western blots. This work also showed that yields of HPV-11 L1 protein in plants could be increased by between 500 and 1000-fold compared to the only previous report [19], despite the use of a native HPV *L1 *gene rather than a plant codon-optimised version. Inoculation of rabbits with plant extract from both types of plants resulted in a weak immune response, and antisera did not react with native HPV-11 L1 VLPs, nor did they neutralise HPV-11 pseudovirion infectivity. As HPV-11 L1 NLS^+ ^VLPs are the closest counterpart to the actual virus it follows that antibodies generated from injection of our plant-derived HPV-11 L1 NLS^- ^protein will not be able to prevent infection and disease. This has serious implications for the further development of plant-derived HPV-11 vaccines.

## Methods

### Cloning of HPV-11 L1 into *Agrobacterium tumefaciens*

Full-length and truncated HPV-11 DNA fragments derived from the *L1 *ORF were amplified from a HPV-11 DNA-positive biopsy sample clone (A-L Williamson, University of Cape Town) using primer pairs 1 and 2 respectively (Table 1), cloned into the pMOSBLUE vector and sequenced. Primer pair 2 was used to exclude the bipartite C-terminal nuclear localisation signal (NLS; **KR**pavskpstap**KRKRTKTKK**) spanning aa 482 – 502. PCR was performed using standard cycling conditions, with an annealing temperature of 55°C. A silent mutation at position 257 (C to T) was found, otherwise this HPV-11 *L1 *sequence does not differ from the prototype HPV-11 *L1 *sequence (GenBank accession number M 14119).

The HPV-11 *L1 *and HPV-11 *L1 NLS*^- ^ORFs were excised and cloned into pUC19 (*Eco*RI/*Sal*I), and thereafter directionally cloned (*Eco*RI/*Hind*III) into plasmid pART7: this placed the *L1 *genes downstream of the CaMV35S promoter and upstream of the octopine synthase gene terminator (*ocs *3'). This cassette was excised (*Not*I) and cloned into the *A. tumefaciens *binary vector pART27 [26]. *A. tumefaciens *C58C1Rif^R ^competent cells were transformed with HPV-11 *L1*/HPV-11 *L1 NLS*^- ^pART27 DNA using a freeze-thaw method [27]. Selection was done on media containing kanamycin (40 μg/ml) and rifampicin (100 μg/ml) at 30°C. Transformants were screened by PCR as described above, using primer pairs 3 (HPV-11 *L1*) and 4 (HPV-11 *L1 NLS*^-^) (Table 1).

### Synthesis of HPV-11 L1 protein in insect cells

The full-length and NLS^- ^*L1 *genes described above were cloned, using standard techniques, into the pFastBac vector (Invitrogen™), transformed into *Escherichia coli *DH10BAC competent cells, and recombinant baculovirus was generated by transfection of the bacmid DNA into *Sf*21 cells (Manufacturer's protocol, Invitrogen™, Carlsbad, California). HPV-11 L1 protein was produced in *Sf*21 cells and virus-like particles (VLPs) were prepared as described [17].

### Transformation of *Arabidopsis thaliana*

*A. thaliana *ecotype Columbia plants were transformed with *A. tumefaciens *constructs (HPV-11 *L1*/HPV-11 *L1 NLS*^-^) by a simplified transformation protocol [28]. Primary bolts were cut off to induce proliferation of secondary bolts, then after 4 days plants were dipped in *A. tumefaciens *cultures containing either pART27 *L1 *or pART27 *L1 NLS*^-^. *A. tumefaciens *cultures were harvested after 2 days and resuspended to OD_600 _= 0.8 in phosphate buffered saline (PBS, 1.47 mM KH_2_PO_4_, 10 mM Na_2_HPO_4_, 2.7 mM KCl, 137 mM NaCl, pH 7.4) containing 5% (w/v) sucrose and surfactant Silwet-77 at a concentration of 0.03% (v/v). Dipped plants were grown at 16/8 hr day/night photoperiod until seeds were harvested. To determine the transformation efficiency and to screen for transformed plants, dry seeds were sterilised in 10% bleach, washed 3 times in water and plated on plant nutrient sucrose medium (PNS) containing kanamycin (250 μg/ml). Germination was allowed to take place under a 16/8 hr photoperiod, at 25°C and 80% relative humidity. Seedlings were left on PNS media until complete development of a mature 4^th ^leaf, before being transplanted to soil.

### Transformation of *Nicotiana tabacum*

The transformation protocol of Horsch *et al*. [29] was used for the generation of transgenic *N. tabacum *plants. Sterilised leaf discs (±1 cm^2^) were dipped into a 48 hr *A. tumefaciens *culture carrying either the HPV-11 *L1 *or HPV-11 *L1 NLS*^- ^construct. Squares were transferred to non-selective co-cultivation media [30] for 72 hrs, follwed by MS regeneration/shooting media with kanamycin (300 μg/ml) and cefotaxim (250 μg/ml) until small shoots appeared from the calli. Shoots with 1 to 2 leaves were transferred to MS root-inducing media with kanamycin (100 μg/ml). Once roots were formed, plants were transferred to soil and grown to maturity with a 16/8 hr photoperiod at 25°C and 80% relative humidity. Flowering plants were self-pollinated and dry seeds screened on media containing kanamycin (250 μg/ml) before putative transgenic seedlings were transferred to soil, once the 4^th ^leaves had grown to maturity.

### Screening of plants for the HPV-11 *L1 *and *L1 NLS*^- ^genes

Plant genomic DNA was extracted from putative transgenic and wild type *A. thaliana *and *N. tabacum *plants using the Dellaporta method [31] and screened by PCR for the HPV-11 *L1 *and HPV-11 *L1 NLS*^- ^genes using primer pairs 3 and 4 (Table 1).

### Analysis of total RNA extracted from transgenic plants

Total RNA was extracted from fresh leaves using TRIzol™ reagent (Life Technologies). Detection of the *L1 NLS*^- ^mRNA was achieved by RT-PCR using the Access RT-PCR system (Promega). Primer pair 5 (Table 1) was used to amplify an internal 300 base pair fragment according to the manufacture's instruction.

### Processing and concentration of transgenic plant material

Entire *A. thaliana *plants and leaves from *N. tabacum *were harvested and homogenised in 1:2 (w/v) cold 0.1 m phosphate/0.5 m NaCl ph 7.4 high salt buffer (HSP buffer). The homogenate was filtered through cheesecloth and centrifuged at 6000 × g for 10 min. 10% (w/v) polyethylene glycol (PEG mw 8000) was added to the supernatant before it was centrifuged at 6000 × g for 20 min. The pellet was resuspended in 1/10^th ^original volume HSP buffer and centrifuged at 6000 × g for 20 min to remove further debris; the remaining supernatant was centrifuged at 100.000 × g for 180 min. The final pellet was resuspended in 1/10^th ^of the previous volume of hsp buffer used.

### Monoclonal antibody characterisation of plant-derived HPV-11 L1 protein

HPV-11 L1 NLS^- ^protein-containing extracts derived from *A. thaliana *and *N. tabacum*, together with non-transgenic plant protein extracts, were characterised by ELISA using a panel of Mabs to HPV-6, HPV-11 and HPV-16 [32], (Table 2). ELISA plates were coated with protein extracts for 1 hr and then blocked in 5% non-fat milk in PBS for 1 hr. Mabs (1:1000) were used to detect the plant-derived antigen for 1 hr. Anti-mouse-alkaline phosphatase conjugated secondary antibody (1:5000, Sigma) was allowed to bind the primary antibody for 1 hr at 37°C. The secondary antibody was detected using p-nitrophenyl phosphate (pNPP, Sigma) and the absorbance measured at 405 nm. All samples were analysed in triplicate to determine the mean absorbance and calculate the respective standard deviation.

### Western blot analysis of plant-derived HPV-11 L1 protein

**5% (v/v) **trichloroacetic acid (TCA) was added to plant protein extracts before being centrifuged at 8000 × g for 20 min at 4°C. The pellet was washed twice in 100% acetone/20 mM HCl before the pellet was resuspended in 25 mM Tris, 2% SDS (1/5^th ^original volume). Plant protein extracts were denatured at 100°C for 10 min in SDS-PAGE gel loading buffer, resolved on a 12% acrylamide gel and transferred onto nitrocellulose membrane by semi-dry electroblotting. L1 protein was detected with Mabs to HPV-6 L1 (H6:C6 and H6:E51, Table 2) at dilutions of 1:1000 and then probed with goat anti-mouse-alkaline phosphatase conjugated secondary antibody at 1:5000. Colorimetric detection was performed with NBT/BCIP tablets (Roche)

### Electron microscopy of HPV-11 L1 protein

*A. thaliana *and *N. tabacum *plant protein extracts and insect cell-derived HPV-11 L1 protein were viewed either directly or after immunotrapping onto carbon-coated copper grids. Protein samples were directly adsorbed onto grids as described [21] or immunotrapped with rabbit-anti-HPV-11 L1 antiserum raised against insect cell-derived HPV-11 L1 VLP (1/50) Grids were washed in H_2_O and stained with 2% uranyl acetate.

Immunogold-labeling of plant-derived L1 protein involved immunotrapping of L1 as described above, probing grids with HPV-11 MAb H11:H3 (1:1000) and then incubating grids with the secondary gold-labeled anti-mouse-conjugated antibody (1:100) (Sigma, 30 nm gold particles) followed by staining with 2% uranyl acetate.

### Immunisation of New Zealand white rabbits

Concentrated *A. thaliana *and *N. tabacum *protein extracts (500 μl) were mixed 1:1 (v/v) with Freund's incomplete adjuvant and injected into New Zealand white rabbits (3 subcutaneous and 1 intramuscular site) on days 1, 28 and 56. Serum collected on days 1, 15, 28, 42, 56 and 70 was analysed by ELISA (1:20 dilution) against insect cell-derived HPV-11 L1 and HPV-11 L1 NLS^- ^VLPs coated onto plates at a concentration of 0.4 μg/100 μl.

### Pseudovirion neutralisation experiments

HPV-11 L1 pseudovirions were generated according to the protocol described by Pastrana *et al*. [33] with plasmids obtained from John Schiller (Laboratory of Cellular Oncology, National Cancer Institute, Bethesda, MD). Sera from inoculated rabbits collected on Day 56 as well as the pre-inoculation sera (Day 1) were evaluated for their capability to neutralise the HPV-11 L1 pseudovirions *in vitro*. Threefold dilutions of all pre- and post-inoculation sera ranging from 1:50 to 1:12,150 were prepared. Sera were incubated with HPV-11 pseudovirions and the resulting secreted alkaline phosphatase (SEAP) content was determined by application of the Great ESCAPE SEAP chemiluminescence kit (BD Clontech) according to the manufacturer's instructions.

## Authors' contributions

TOK participated in the design of the study, carried out most of the experimental work and drafted the manuscript. IIH participated in the design of the study, coordinated animal experiments and virus neutralisation assays and helped drafting and revising the manuscript. NDC supplied the monoclonal antibodies utilised in the study and participated in experimental design. EPR conceived the study, participated in its design and helped to revise the manuscript. All authors read and approved the final version.
